# Primary headache disorders: Five new things

**DOI:** 10.1212/CPJ.0000000000000654

**Published:** 2019-06

**Authors:** Peter J. Goadsby

**Affiliations:** NIHR-Wellcome Trust King's Clinical Research Facility and SLaM Biomedical Research Centre, King's College London, UK; and Department of Neurology, University of California, San Francisco.

## Abstract

**Purpose of review:**

To review 5 new areas in primary headache disorders, especially migraine and cluster headache.

**Recent findings:**

Calcitonin gene-related peptide (CGRP) receptor antagonists (gepants—rimegepant and ubrogepant) and serotonin 5-HT_1F_ receptor agonists (ditans—lasmiditan) have completed phase 3 clinical trials and will soon offer novel, effective, well-tolerated nonvasoconstrictor options to treat acute migraine. CGRP preventive treatment is being revolutionized after the licensing of 3 monoclonal antibodies (MABs), erenumab, fremanezumab, and galcanezumab, with eptinezumab to follow, especially designed for migraine; they are effective and well tolerated. For patients seeking a nondrug therapy, neuromodulation approaches, single-pulse transcranial magnetic stimulation, noninvasive vagus nerve stimulation (nVNS), and external trigeminal nerve stimulation, represent licensed, well-tolerated approaches to migraine treatment. For the acute treatment of episodic cluster headache, nVNS is effective, well tolerated, and licensed; nVNS is effective and well tolerated in preventive treatment of cluster headache. The CGRP MAB galcanezumab was effective and well tolerated in a placebo-controlled trial in the preventive treatment of episodic cluster headache. Sphenopalatine ganglion stimulation has been shown to be effective and well tolerated in 2 randomized sham-controlled studies on chronic cluster headache. Understanding the premonitory (prodromal) phase of migraine during which patients experience symptoms such as yawning, tiredness, cognitive dysfunction, and food cravings may help explain apparent migraine triggers in some patients, thus offering better self-management.

**Summary:**

Headache medicine has made remarkable strides, particularly in understanding migraine and cluster headache in the past 5 years. For the most common reason to visit a neurologist, therapeutic advances offer patients reduced disability and neurologists a rewarding, key role in improving the lives of those with migraine and cluster headache.

Primary headache disorders, such as migraine and cluster headache, are the most common reasons, for which patients seek neurologic advice, and every year affect nearly 3 billion people^1^; thus, any new therapy would be of broad interest. It was a challenge to adopt a marmoreal attitude to a single therapeutic advance, triptans, serotonin 5-HT_1B/1D_ receptor agonists, when they came.^2^ Thirty years later, and certainly since the last of this series of articles in 2011, 4 new things have happened in therapeutics alone; exciting seems a trite description when thinking about headache medicine in 2019. Because new treatments are affecting, or will affect, neurologists in 2019, I will cover them in detail. An emerging area of clinical neuroscience is the study of nonheadache phases of migraine, notably the premonitory (prodromal) and postdromal phases. They provide insights into mechanisms that can be used in clinical practice. Many other areas in the primary headache disorders have advanced in the past decade, so readers interested in pathophysiology of migraine^3^ or cluster headache,^4^ headache classification,^5^ or the genetics of headache^6^ are referred to recent reviews wherein wallahs riff.

New acute therapies for migraine attacks, gepants, calcitonin gene-related peptide (CGRP) receptor antagonists, and ditans, serotonin 5-HT_1F_ receptor agonists, offer novel approaches to the treatment. Preventive therapy targeting the CGRP pathway using monoclonal antibodies (MABs) or small molecule CGRP receptor antagonists offers a remarkable clinical advance. Neuromodulation approaches to migraine, both acute and preventive, provide nondrug options for physicians to deploy and patients to take benefit from. Being important for patients and neurologists, therapeutic developments in medicines and neuromodulation are underway for cluster headache. The therapeutic developments are framed as comments from a patient, and replies that clinicians may now make. Citations for data concerning these developments are given in table. Reformulation of triptans is covered here; certainly, more efficient delivery systems, such as a permeation enhancer for nasal sumatriptan^7^ or an adhesive dermally applied microarray for zolmitriptan,^8^ offer new avenues for medicines that are widely used and well liked. Here, newer approaches that neurologists may be less familiar with have been chosen for a more detailed study. Understanding the premonitory phase of migraine affords the clinician expanded dimensions in the clinical history that aid diagnosis and offer an opportunity to demonstrate to patients a deeper understanding of their presentation and disability.

**Table:**
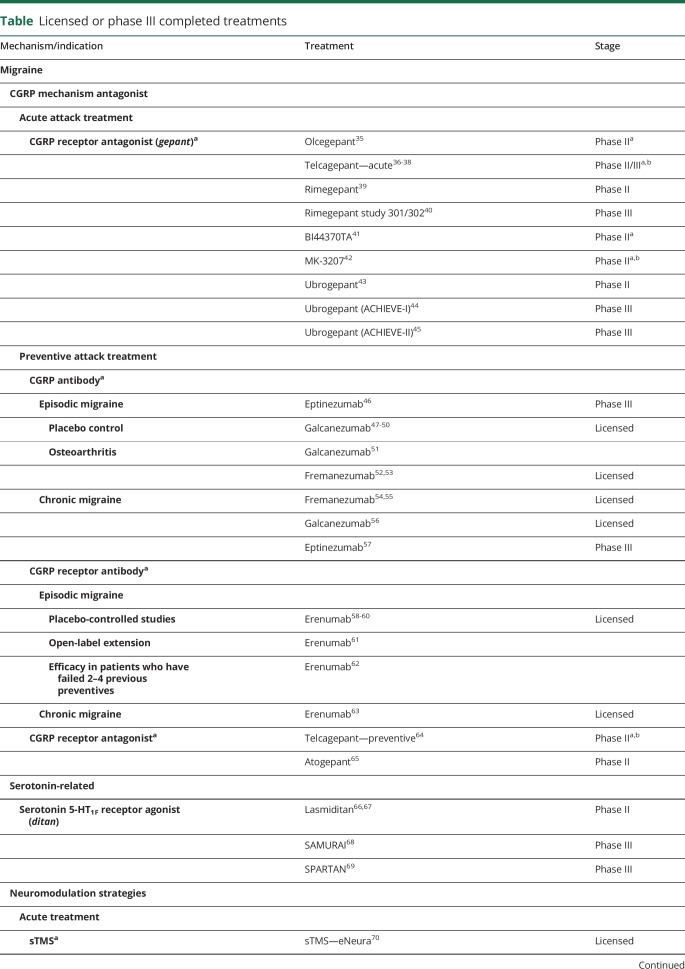

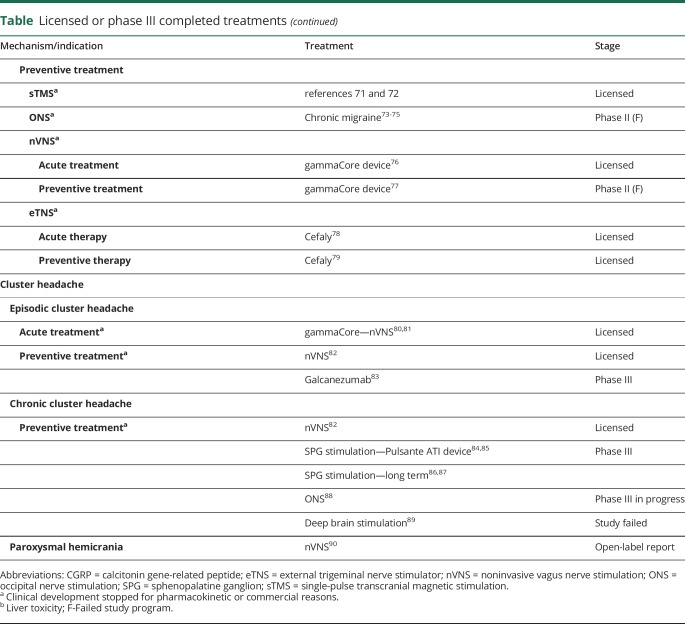
Licensed or phase III completed treatments

## What can you give me for my migraine? I cannot take triptans. *Gepants or Ditans*?

Triptans were developed based on the hypothesis that the pain of migraine was due to dilation of extracerebral cranial vessels.^9^ Although undoubtedly useful and effective in suitable patients, triptans have 3 major limitations: not all patients respond, not all patients tolerate the medicines,^2^ and for some patients with cardiovascular or cerebrovascular disease they are contraindicated.^10^ Although the neural, nonvasoconstrictor effect of triptans has been postulated for 2 decades,^11^ the vasodilator hypothesis of migraine took some time to be shown as inadequate.^12^ Readers should not consider that just anything works for migraine; there is a long list of failed approaches, including substance P/neurokinin 1 receptor antagonists, plasma protein extravasation inhibitors, and TRPV1 receptor antagonists,^13^ which offer comfort to the positive outcomes.

### Gepants—CGRP receptor antagonists

CGRP was shown to be important for migraine in translational studies in the late 1980s.^14–16^ CGRP can trigger migraine,^17^ and blockade of the canonical CGRP receptor^18^ is effective in the treatment of acute migraine (table). Gepants have no active vasoconstrictor effect. Six gepants have been tested, and each was effective in the acute treatment of migraine. Two were terminated during development because of hepatic concerns, which now seem firmly based on metabolites not on the CGRP mechanism. Two CGRP receptor antagonists, rimegepant and ubrogepant, have completed phase III studies. They are both effective at the primary endpoints of being free of pain for 2 hours and most bothersome symptom (MBS), as currently recommended by the US FDA. The latter endpoint is new: patients are asked to nominate which of nausea, photophobia, or phonophobia bothers them the most during the migraine and which symptom is absent for 2 hours. Interestingly, photophobia has dominated in all phase III studies as the MBS. Gepants are remarkably well tolerated with only a few percentage points or less excess of nausea or somnolence reported above placebo. There have been no cardiovascular or cerebrovascular concerns, as would be expected from the mechanism, given peptide redundancy in the CGRP class, nor have liver enzyme issues emerged.

#### Are gepants potentially disruptive therapies?

Across the studies, there is remarkable consistency in the population effect: about 20% of migraineurs are pain free at 2 hours. The population effect is smaller than triptans, or ditans, where about one-third of migraineurs are pain free at 2 hours. From an intrapatient perspective, in pain-free patients, they are just as pain free as they would be on a triptan or a ditan; they may be better off given the tolerability is improved. Moreover, given the preventive data with the CGRP pathway, both from MABs and gepants used preventively, it seems highly likely that medication overuse will simply not be a problem since it would seem the more often a gepant is dosed, the less migraine the patient has. Studies on gepants suggest that the dichotomous view of acute therapies vs preventive therapies for migraine is artificial because the CGRP pathway can be engaged for either purpose. Perhaps the most disruptive aspect of this new class is that biology-driven developments can be targeted at the clinical need not constrained as either acute or preventive.

### Ditans—serotonin 5-HT_1F_ receptor agonists

Not all triptans were created pharmacologically similar; although sumatriptan is active at the 5-HT_1F_ receptor, rizatriptan is not.^19^ Does activation of the 5-HT_1F_ receptor can inhibit trigeminal neuronal activity without any vascular effect? The fact is that there are now 4 positive randomized placebo-controlled trials including 1 with intravenous administration and no cardiovascular adverse events, establishes that a purely neural antimigraine effect works. About one-third of patients in the phase III studies are pain free at 2 hours, and the MBS endpoint was met (table). Dizziness was reported in 16% and 13% of patients on 200 and 100 mg, respectively, and in 3% in the placebo arm (SAMURAI; table). It is clear that this medicine acts centrally in some parts. There will be a clinical balance to strike; vertigo is a very common sensation in migraine. It is not yet clear from the studies whether dizziness was a price to pay for headache resolution or unrelated; in general, no side effect of triptans seems to predict efficacy reliably, if that is any guide. For patients who have failed to respond or have contraindications to triptans, ditans will probably be available. Unsurprisingly, given their presynaptic action in contrast to the postjunctional/synaptic action of gepants, more patients respond. Perhaps also not surprising is an emerging broad principle that more specific treatments in terms of targets yield generally fewer side effects. Presynaptic approaches block the release of multiple transmitters, so they are generally more likely to work across a population and more likely to have some side effects, whereas postsynaptic, single transmitter targets broadly treat a smaller population and seem generally better tolerated. Both will be welcome additions to our therapeutic options in the future.

## I have too many migraines: *I will give you a migraine preventive*

About 40% of patients with episodic migraine,^20^ i.e., affected less than 15 days a month, and probably all patients with chronic migraine should be offered preventive therapy. If one considers compliance as an indication, 80% or more of patients with chronic migraine will be nonadherent to treatment at 12 months.^21^ Some part of this must be clinical improvement, certainly not all. Current migraine preventive therapies provide an eclectic mechanistic background: hypertension, epilepsy, depression, neurotoxin or neutriceutical supplements. Each dragooned into migraine without a theme or particular insight into the underlying condition. How often has the reader said in response to the patient comment above: I will give you X—it was developed for Y but works in migraine—*don't worry*…. CGRP pathway blockers provide the first mechanism-specific, migraine-bespoke preventive therapies for the condition. Three MABs are now licensed: 2 to the peptide (galcanezumab and fremanezumab), with 1 to come (eptinezumab), and 1 to the canonical CGRP receptor (erenumab). I emphasize canonical here, the calcitonin receptor (CTR)-like receptor/receptor activity modifying peptide 1 (RAMP-1), CGRP receptor, as opposed to the amylin, CTR/RAMP-1, receptor that ex vivo can be activated by CGRP.^22^ This will hopefully be resolved soon in vivo. The MABs are effective in episodic migraine from 4 to 14 days and chronic migraine (table). They are extremely well tolerated with dropout rates a few percent compared with, for example, the topiramate development program at 30% or more. Injection site discomfort is the common side effect in the class, and constipation has emerged greater than the *softer* numbers in the controlled trials. There have been no cardiovascular issues, no liver enzyme issues, and predictably, as MABs, no drug interactions. Of the responders who continue on therapy for a year, a remarkable 40% will have a 75% or more reduction in migraine days, while approximately 25% will be migraine free (table). Moreover, retrospective and prospective studies demonstrate that patients who have failed up to 4 previous preventives are still likely to respond to these medicines; this is naturally the group clinicians will be treating early in their experience (table). Safety in medium-term use, up to 3 years, is reassuring; notably a study of galcanezumab on osteoarthritis was negative in terms of efficacy, no new adverse events appeared in this somewhat older cohort (table). Not all patients respond because it is likely that CGRP is at least not so important in all patients and perhaps even varies in pathophysiologic significance within patients over time. It is obvious, even jejune, to say we do not know what we cannot know: what will happen in the long term for both efficacy and side effects. No doubt—despite redundancy, CGRP is important in some individuals in ways that we are yet to understand. As we know that large numbers of migraineurs have now been exposed to, many have performed very well for the short term and few have had tolerability problems associated with current therapies. How the MABs will be positioned in chronic migraine therapy with regard to onabotulinum toxin type A will take time to resolve. Given the large unmet need in migraine prevention, it is easy to see the treated pool increase, and the need for neurologic expertise expanded, with growth in all the tools we have.

## I do not want another pill for my migraine: *I have nonpill options*

This refrain will be familiar to every practicing neurologist; it is borne of genuine and reasonable frustration with pharmacologic therapies that have not worked or resulted in a plethora of side effects. Devices with near-zero morbidity, and approved by the US Food and Drug Administration (FDA), can now be deployed for both acute and preventive treatment of migraine. Single-pulse transcranial magnetic stimulation (sTMS) delivers a nominal 0.9T pulse over cranial bone that has been shown in experimental settings to alter dural nociceptive trigeminothalamic activation.^23^ This device is approved by the FDA for both acute and preventive treatments of migraine (table). Given the established safety of MRI during pregnancy,^24^ it can be extremely useful in clinical practice as well. Noninvasive vagus nerve stimulation (nVNS) delivers five 5 kHz pulses at 25 Hz for 120 seconds; it has been shown to alter nociceptive trigeminovascular transmission in experimental settings.^25^ The device is FDA approved for acute treatment of migraine and well tolerated in practice (table). Stimulation of the supraorbital region with an external trigeminal nerve stimulator (eTNS) is FDA approved for both acute and preventive treatments of migraine (table). The device delivers a 100-Hz stimulation to the forehead bilaterally with a cutaneous adhesive device, which in preventive use alters brain activity in the orbitofrontal and rostral anterior cingulate cortices.^26^ Finally, it should be noted that there are 3 negative studies for occipital nerve stimulation (ONS) in migraine (table). It can be argued, based on the data, that ONS should only be considered after noninvasive neuromodulation, at least 3 standard oral preventives of different classes, a CGRP peptide and a receptor MAB, and a course of intravenous dihydroergotamine in the hospital,^27^ including any medication overuse withdrawal (not listed here in a particular order).

## Please help me; I have cluster headache: *Yes, I can do that*

This is a simple refrain of deep suffering that demands our attention. Clinicians will immediately understand why therapeutic advances are important here. Current standard of care for the acute treatment of cluster headache includes inhaled oxygen, intranasal zolmitriptan or sumatriptan, and injectable sumatriptan.^4^ nVNS has now 2 positive sham-controlled studies (table) and is FDA approved for the acute treatment of attacks in episodic cluster headache; the device was not effective in chronic cluster headache. The device is also approved for the preventive treatment of both episodic and chronic cluster headache. Although not FDA approved, nVNS has been reported to be effective in paroxysmal hemicrania and hemicrania continua—indomethacin-sensitive trigeminal autonomic cephalalgias that can be very difficult to manage when patients develop GI complications of indomethacin (table). Based on good translational studies showing CGRP is elevated in acute cluster headache^28^ and CGRP can trigger attacks in patients in bout,^29^ galcanezumab, a CGRP MAB, has been shown in a randomized placebo-controlled trial to be effective in the preventive management of episodic cluster headache. Again, it is of interest that no effect was seen in chronic cluster headache (table). For clinicians who manage patients with chronic cluster headache, 2 studies have recently reported the efficacy of sphenopalatine ganglion (SPG) stimulation with the Pulsante device against sham-controlled devices (table). SPG stimulation is effective in both treating acute attacks and reducing the frequency of attacks in the long term (table). The device is well-tolerated and, importantly, was designed for the purpose. ONS is currently being evaluated for cluster headache in a controlled trial (table) and thus should be viewed as experimental. Deep brain stimulation has been shown in a sham-controlled trial to be ineffective (table); its use, if any, should be in experimental settings only as a last resort.

## Should I avoid chocolate to prevent my migraine: *No!*

One can hear a groan as the reader recalls the very many times this and questions like these being asked—the apparent holy grail of trigger avoidance. The premonitory (prodromal) phase of migraine consists of a period of hours or days before the headache phase in which symptoms, such as tiredness, yawning, neck discomfort, mood change, cognitive impairment (a brain fog), thirst, polyuria, or cravings for sweet or salty foods, begin.^5^ Observation and clinical history taking will also show that cranial autonomic symptoms, such as lacrimation, nasal congestion, or aural fullness,^30^ can occur, as can nausea, photophobia, or phonophobia. Indeed, these symptoms can all persist in the headache phase. The premonitory phase is marked by functional imaging changes in the region of the hypothalamus.^31,32^ Given the known roles and interactions between satiety mechanisms, nociception, and the hypothalamus,^3,33^ one might reformulate the trigger search question. Perhaps, for example, in some patients, they have a hypothalamic-driven consumption of sweet foods 6–8 hours before a migraine; they succumb to the desire, and they suffer a migraine. They make the completely correct association of migraine with chocolate consumption for a completely wrong reason because their attack has already started. Similarly, light sensitivity does not trigger migraine but warns of its onset. Being aware of premonitory symptoms then becomes a management strategy for patients that affords them some degree of predictability. The neurologic history begins to both build confidence in the therapeutic relationship and, through pathophysiologic knowledge, the neurologist is adding a clinical value to the migraineur.

## Conclusion

Although primary headache disorders have their challenges, such as the epistemological limits of self-reference in the current system of headache classification^5^ that only biology will resolve, the lack of new treatments for tension headache, or the inadequate funding by NINDS and other agencies to headache medicine over the past 30 years, neurologists for reasons of interest or utility can embrace new therapeutics. Being able to offer a migraine patient a migraine bespoke preventive, rather than a hand-me-down from another condition, demonstrates an apophthegm that biological research works. Deploying the new acute and preventive therapies or neuromodulation approaches, and indeed explaining advances in pathophysiology, such as understanding the premonitory phase to shed light on putative migraine triggers,^34^ underscores the value of neurology to patients and broadly to society. This is an exciting time to be in medicine and the most exciting period to be in headache medicine.
